# Crystal structure of 3-bromo-2-hy­droxy­benzoic acid

**DOI:** 10.1107/S2056989015007331

**Published:** 2015-04-22

**Authors:** Gerhard Laus, Volker Kahlenberg, Thomas Gelbrich, Sven Nerdinger, Herwig Schottenberger

**Affiliations:** a University of Innsbruck, Faculty of Chemistry and Pharmacy, Innrain 80, 6020 Innsbruck, Austria; b University of Innsbruck, Institute of Mineralogy and Petrography, Innrain 52, 6020 Innsbruck, Austria; c Sandoz GmbH, Biochemiestrasse 10, 6250 Kundl, Austria

**Keywords:** crystal structure, hydrogen bonding, structural systematics, XPac, salicylic acid derivative

## Abstract

Mutual carbox­yl–carboxyl O—H⋯O hydrogen bonds link the mol­ecules of the title compound, C_7_H_5_BrO_3_, into centrosymmetric dimers which display a central *R*_2_^2^(8) ring motif. In addition, there is an intra­molecular hydrox­yl–carboxyl O—H⋯O inter­action present. A comparison with the crystal structures of 59 other substituted derivatives of salicylic acid shows that both the centrosymmetric carbox­yl–carboxyl O—H⋯O dimer and the stacking mode of mol­ecules along the short *a* axis observed in the title structure are frequent packing motifs in this set.

## Abstract 2

Centrosymmetric dimers with a central 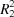
(8) ring motif are formed as a result of (carbox­yl)O—H⋯O(carbox­yl) hydrogen bonds. In addition, there is an intra­molecular (hydrox­yl)O—H⋯O(carbox­yl) hydrogen-bonding inter­action.

## Chemical context   

Substituted derivatives of salicylic acid are widely used in organic synthesis and can be biologically active. Members of this class have served as model compounds for studies of crystal polymorphism (Sarma *et al.*, 2010 ▸; Braun *et al.*, 2011 ▸), the stability of hydrogen bonds (Bawa *et al.*, 2004 ▸; Adam *et al.*, 2010 ▸) or for systematic investigations of crystal-packing relationships (Montis & Hursthouse, 2012 ▸). The title compound is used in the synthesis of 7-bromo­benzoxazolin-2-one (Laus *et al.*, 2011 ▸), which is an inter­mediate in the synthesis of bifeprunox, an experimental drug for the treatment of psychiatric disorders such as schizophrenia (Zwier *et al.*, 2005 ▸; Eijgendaal *et al.*, 2006 ▸).
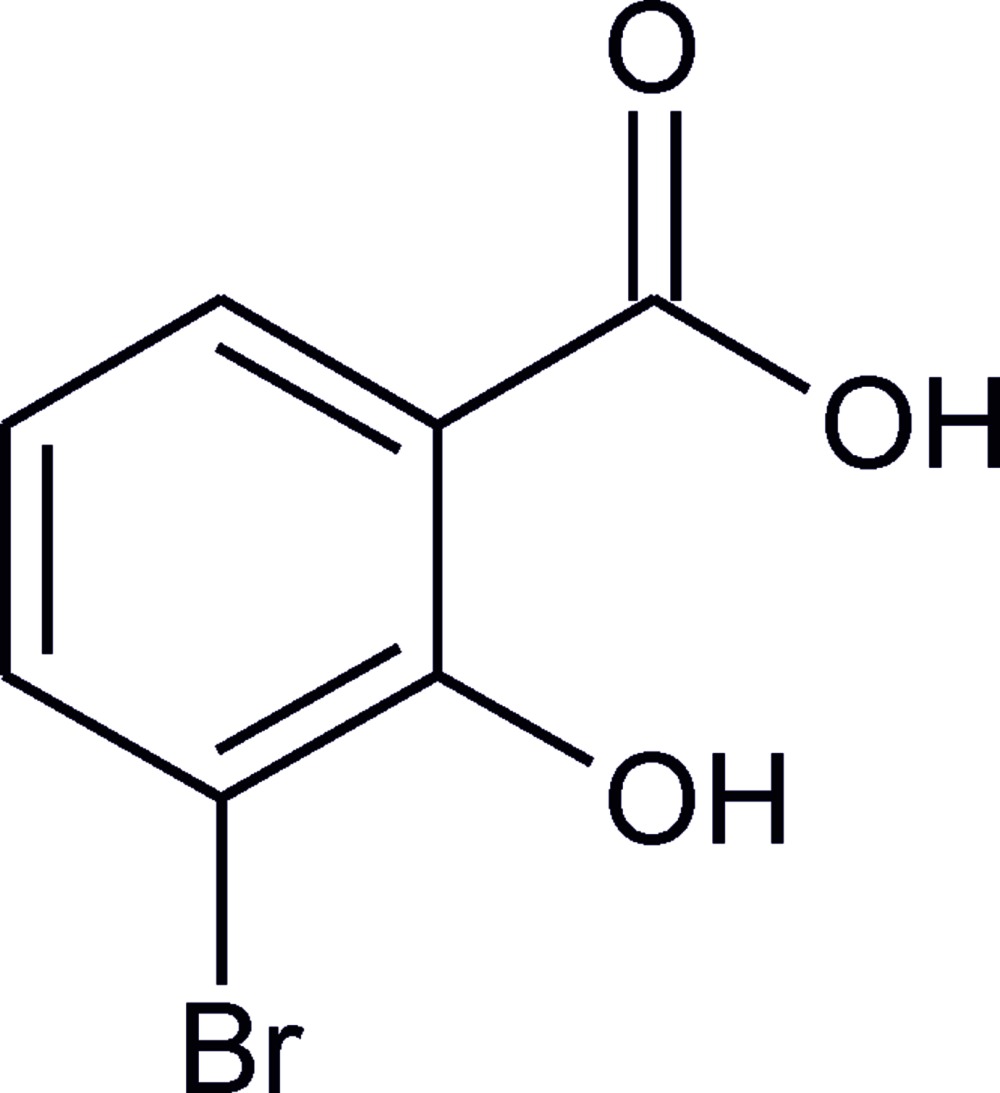


## Structural commentary   

**Figure 1:**
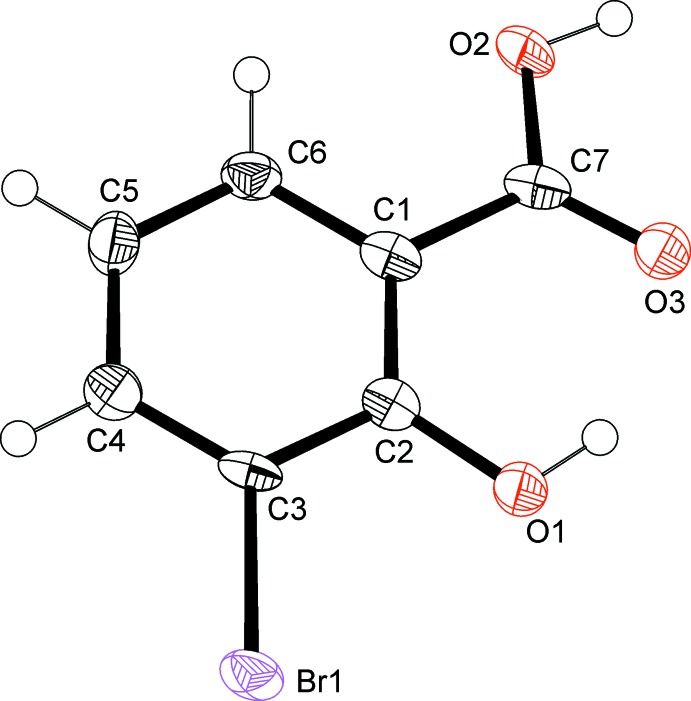
The mol­ecular structure of the title compound, drawn with displacement ellipsoids at the 50% probability level. H atoms are drawn as spheres of arbitrary size.

The mol­ecule is almost planar (Fig. 1 ▸). The plane defined by the non-H atoms of the carboxyl group is slightly twisted by 4.7 (4)° to the mean plane of the phenyl ring. An intra­molecular hydrogen bond, O1—H1⋯O3 (Table 1 ▸), connects the hydroxyl group bonded to C2 with the carboxyl group at C1.

## Supra­molecular features   

**Figure 2:**
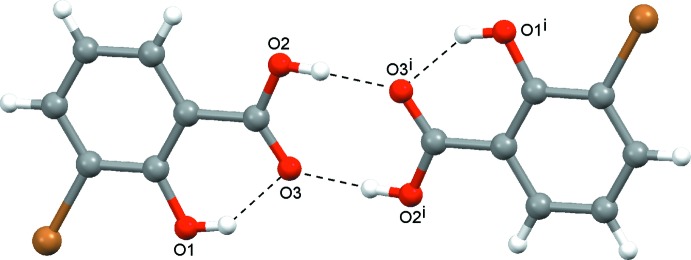
Centrosymmetric dimer with a central 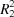
(8) ring motif. Dashed lines indicate hydrogen bonds. [Symmetry code: (i) −*x* + 2, −*y*, −*z* + 1.]

**Figure 3:**
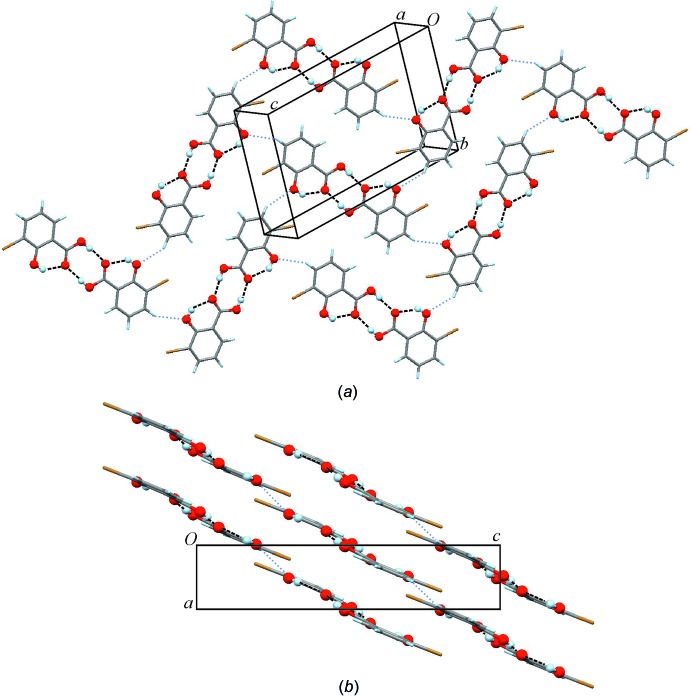
(*a*) Mol­ecular packing in the (10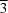) plane. Dashed lines indicate O—H⋯O hydrogen bonds; C4—H4⋯O1(−*x* + 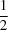, *y* + 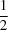, −*z* + 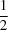) contacts are indicated by thin dotted lines. Similar arrangements are present in the crystal structures of 3,5-Br and 3,5-Cl (supra­molecular construct S2). (*b*) Three corrugated sheets stacked in the *a-*axis direction.

Neighbouring mol­ecules are linked to one another by a two-point O2—H2⋯O3(−*x* + 2, −*y*, −*z* + 1) connection involving a pair of anti­parallel inter­actions between their carboxyl groups (Fig. 2 ▸, Table 1 ▸). The resulting centrosymmetric dimer is described by the graph set 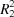
(8) (Etter *et al.* 1990 ▸; Bernstein *et al.*, 1995 ▸). In the crystal structure, the dimers, which are essentially planar units, assemble into slightly corrugated sheets which lie parallel to the (10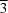) plane. A sheet of this kind contains a short C4—H4⋯O1(−*x* + 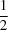, *y* + 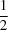, −*z* + 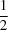) contact (H⋯O = 2.57 Å, C—H⋯O = 145.7°) (Fig. 3 ▸ *a*) which involves the hydroxyl group at C2. The sheets are stacked in a parallel fashion in the *a*-axis direction with an inter­sheet separation of 3.798 (4) Å which corresponds to the length of this axis.

## Database survey   

**Figure 4:**
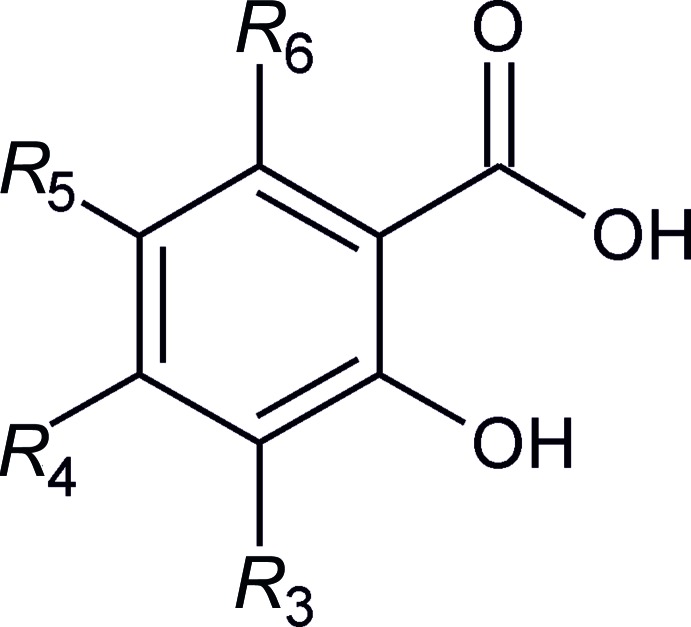
Scheme showing the general composition of substituted derivatives of salicylic acid, the crystal structures of which were compared in this study.

A systematic study of packing motifs present in 24 crystal structures of monosubstituted derivatives of salicylic acid has previously been published by Montis & Hursthouse (2012 ▸), who also proposed a nomenclature where a substituent *R^n^* at a ring position *n* (*n* = 3, 4, 5 or 6) is encoded *n-R^n^* (Fig. 4 ▸; *R^n^* ≠ H). The title compound of the present study is denoted 3-Br in this system. Our own survey of the Cambridge Structural Database (version 5.25; Groom & Allen, 2014 ▸) revealed 59 unique crystal structures of salicylic acid derivatives, listed in Table S1 of the Supporting information, which are close structural analogues of 3-Br. This set includes several polysubstituted derivatives as well as multiple-component crystals and crystal structures containing potential hydrogen bond donor and acceptor sites in addition to those of the 1-hydroxyl and 2-carboxyl groups.

In order to establish the possible existence of geometrically similar substructure units, pairwise *XPac* comparisons (Gelbrich & Hursthouse, 2005 ▸) were carried out between the crystal structure of 3-Br on one hand and each of the other 59 salicylic acid derivatives on the other. Analogous to the study by Montis & Hursthouse (2012 ▸), the underlying calculations were based on the comparison of inter­molecular geometrical parameters generated from the ten non-H atomic positions of the salicylic acid mol­ecular core (C_7_O_3_) which is present in all compounds of the set. A qu­anti­tative descriptor, the dissimilarity index *x*_10_ (Gelbrich *et al.*, 2012 ▸), was calculated for each common supra­molecular construct (SC) identified. In general, a larger *x*_10_ value indicates a lower degree of similarity and an *x*_10_ value significantly higher than 10 is consistent with a situation where the fundamental features of a 3-Br substructure unit are also present in a second crystal, albeit with considerable geometrical modifications.

41 structures of the investigated set, including 3-Br, contain _(carbox­yl)_O—H⋯O_(carbox­yl)_ hydrogen-bonded dimers with a central 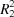
(8) ring motif (Fig. 2 ▸). All of the dimers are centrosymmetric, except for that of 3-COOH (Mereiter *et al.*, 2001 ▸). In the latter structure, the 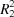
(8) ring motif is inter­sected by a glide plane and connects the 2-carboxyl group of one mol­ecule with the 3-carboxyl group of the other so that an hydrogen-bonded chain structure is formed. Owing to the rigidity of the aromatic ring and the limited rotational flexibility about the C1—C7 bond, all 40 centrosymmetric dimers adopt approximately the same geometry, and the corres­ponding *x*_10_ values are smaller than 12 (Table S1 of the Supporting information). In keeping with the nomenclature introduced by Montis & Hursthouse (2012 ▸), we denote this dimer SC A0.

**Figure 5:**
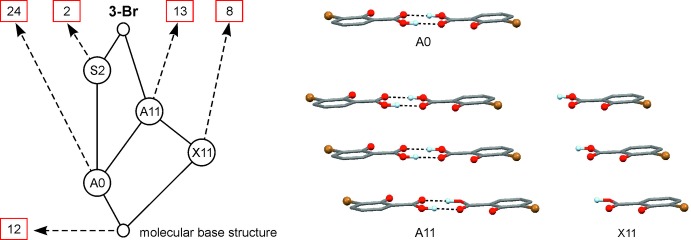
(*Left*) Tree diagram illustrating the packing relationships between 3-Br and other substituted derivatives of salicylic acid. A number in a box indicates the number of crystal structures for which a given SC (A0, X11, A11 and S2) is the largest common SC with 3-Br. (*Right*) Instances of the SCs A0, X11, A11 in 3-Br.

A one-periodic SC, denoted X11 by Montis & Hursthouse (2012 ▸), describes the stacking of 3-Br mol­ecules along the shortest crystallographic axis [*a* axis; Fig. 5 ▸(*right*)]. We have identified another 21 examples of the same stacking mode (Table S1 of the Supporting Information) and the 13 best matches with *x*_10_ > 12 are listed in Table 2 ▸. In this latter subset, the length of the stacking vector varies from 3.67 to 3.98 Å. Moreover, the eleven structures listed in the top section of Table 2 ▸ also contain a centrosymmetric dimer so that their common SC is actually a stack of dimers [denoted A11; Fig. 5 ▸(*right*)].

Other noteworthy packing relationships exist between 3-Br and the structures of 3,5-Br (XISGEM; Liu *et al.*, 2008 ▸) and 3,5-Cl (WECXAE; Gao *et al.*, 2005 ▸). These are based on the sheet structure which lies parallel to (10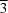) in the 3-Br crystal and is depicted in Fig. 3 ▸ *a*. The corresponding *x*_10_ values of 11.8 and 12.4 for this 2-periodic SC [denoted S2 in Fig. 5 ▸(*left*)] indicate a relaxed form of geometrical similarity, which is consistent with the accommodation of additional halogen substituents in the planes of 3,5-Cl and 3,5-Br. Moreover, the short C4—H4⋯O1 contact found in 3-Br (see above) is replaced by other close contacts in the S2 instances of 3,5-Br and 3,5-Cl. Table S2 of the Supporting information contains the corresponding crystallographic parameters associated with SC S2. A graphical overview of the packing relationships involving 3-Br and their inter­dependencies is given in Fig. 5 ▸(*left*).

## Synthesis and crystallization   

The title compound was prepared from 5-sulfosalicylic acid by bromination, followed by desulfonation in hot phospho­ric acid and, finally, purification by steam distillation, as described by Meldrum & Shah (1923 ▸). Single crystals were obtained by recrystallisation from hot water.

^1^H NMR (DMSO-*d*_6_, 300 MHz): 6.87 (*t*, *J* = 7.9 Hz, 1H), 5.3 (*br*, 1H), 7.80 (*d*, *J* = 7.9 Hz, 2H), 11.5 (*br*, 1H) p.p.m. ^13^C NMR (DMSO-*d*_6_, 75 MHz): 110.1, 114.3, 120.2 (CH), 129.7 (CH), 138.5 (CH), 157.6, 171.6 p.p.m. ^1^H NMR (CDCl_3_, 300 MHz): 6.85 (*t*, *J* = 7.9 Hz, 1H), 7.79 (*dd*, *J* = 7.9 and 1.6 Hz, 1H), 7.92 (*dd*, *J* = 7.9 and 1.6 Hz, 1H), 11.07 (*s*, 1H) p.p.m. ^13^C NMR (CDCl_3_, 75 MHz): 111.8, 112.8, 120.7 (CH), 130.6 (CH), 140.5 (CH), 159.0, 174.1 p.p.m. IR (neat): 2855, 2526, 1653, 1603, 1428, 1298, 1243, 1153, 851, 744, 677, 469 cm^−1^.

## Refinement   

Crystal data, data collection and structure refinement details are summarized in Table 3 ▸. Positions of hydrogen atoms bonded to carbon atoms were generated in idealized geometries using a riding model and their displacement parameters were set to *U*_iso_(H) = 1.2 *U*_eq_(C). The H atoms attached to O were identified from difference Fourier maps and their positions refined with restrained distances [O—H 0.86 (2) Å] and their isotropic thermal displacement parameters were refined freely.

## Supplementary Material

Crystal structure: contains datablock(s) I. DOI: 10.1107/S2056989015007331/wm5143sup1.cif

Structure factors: contains datablock(s) I. DOI: 10.1107/S2056989015007331/wm5143Isup2.hkl

Supporting information file. DOI: 10.1107/S2056989015007331/wm5143Isup3.pdf

Supporting information file. DOI: 10.1107/S2056989015007331/wm5143Isup4.cml

CCDC reference: 1059331

Additional supporting information:  crystallographic information; 3D view; checkCIF report

## Figures and Tables

**Table 1:** Hydrogen-bond geometry (, )

*D*H*A*	*D*H	H*A*	*D* *A*	*D*H*A*
O1H1O3	0.85(2)	1.88(4)	2.604(4)	142(5)
O2H2O3^i^	0.87(2)	1.80(2)	2.664(4)	172(6)

Symmetry code: (i) 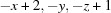.

**Table 2:** One-dimensional packing relationships between 3-Br and other derivatives of salicylic acid, based on the stacking of either individual molecules (X11) or dimers (A11) along the short crystallographic axis and identified with *XPac*

Compound	SC*^*a*^*	*x*_10_ *^*b*^*	*d* ()*^*c*^*	CSD code	reference
3-Br	A11	-	3.80	-	This work
5-F	A11	4.2	3.82	ABENEB	Choudhury Guru Row (2004 ▸)
5-COOH	A11	4.8	3.68	OJICEP	Cox Murphy (2003 ▸)
3,4-OH0.25H_2_O	A11	5.6	3.73	LAPZUZ	Li *et al.* (2012 ▸)
5-OMe	A11	5.6	3.98	VAXZUR	Montis Hursthouse (2012 ▸)
5-Cl	A11	7.1	3.71	VABVAX01	Montis Hursthouse (2012 ▸)
4-Cl	A11	9.2	3.72	VAXYAW	Montis Hursthouse (2012 ▸)
5-NO	A11	9.5	3.67	NTSALA	Talberg (1977 ▸)
4-NH_2_	A11	10.3	3.73	AMSALA02	Montis Hursthouse (2012 ▸)
4-OH	A11	10.7	3.69	ZZZEEU04	Parkin *et al.* (2007 ▸)
4-Me	A11	11.5	3.87	VAXYIE	Montis Hursthouse (2012 ▸)
5-ACMH_2_O	X11	2.2	3.75	VAXYOK	Montis Hursthouse (2012 ▸)
5-CHO	X11	5.6	3.78	UJOFEF	Lu *et al.* (2010 ▸)
3-CHOH_2_O	X11	11.1	3.72	JOHXEJ	Claude *et al.* (1991 ▸)

Notes: (*a*) the largest supramolecular construct which a crystal has in common with that of 3-Br; (*b*) *XPac* dissimilarity index computed from intermolecular geometrical parameters which were calculated using the ten non-H atomic positions of the common salicylic acid molecular fragment; (*c*) the length of the X11 stacking vector.

**Table 3:** Experimental details

Crystal data	
Chemical formula	C_7_H_5_BrO_3_
*M*_r_	217.02
Crystal system, space group	Monoclinic, *P*2_1_/*n*
Temperature (K)	173
*a*, *b*, *c* ()	3.7978(4), 10.5567(6), 18.0366(10)
()	90.208(7)
*V* (^3^)	723.12(10)
*Z*	4
Radiation type	Mo *K*
(mm^1^)	5.63
Crystal size (mm)	0.32 0.16 0.08

Data collection	
Diffractometer	Agilent Xcalibur (Ruby, Gemini ultra)
Absorption correction	Multi-scan (*CrysAlis PRO*; Agilent, 2012 ▸)
*T*_min_, *T*_max_	0.094, 1
No. of measured, independent and observed [*I* > 2(*I*)] reflections	4627, 1594, 1309
*R*_int_	0.040
(sin /)_max_ (^1^)	0.684

Refinement	
*R*[*F* ^2^ > 2(*F* ^2^)], *wR*(*F* ^2^), *S*	0.040, 0.100, 1.08
No. of reflections	1594
No. of parameters	108
No. of restraints	2
H-atom treatment	H atoms treated by a mixture of independent and constrained refinement
_max_, _min_ (e ^3^)	1.19, 0.79

Computer programs: *CrysAlis PRO* (Agilent, 2012 ▸), *SUPERFLIP* (Palatinus Chapuis, 2007 ▸), *SHELXL2014* (Sheldrick, 2015 ▸), *ORTEP-3 for Windows* and *WinGX* (Farrugia, 2012 ▸), *Mercury* (Macrae *et al.*, 2006 ▸), *XP* (Sheldrick, 2008 ▸) and *publCIF* (Westrip, 2010 ▸).

## supplementary crystallographic information

### Crystal data

**Table:**

C_7_H_5_BrO_3_	*F*(000) = 424
*M**_r_* = 217.02	*D*_x_ = 1.993 Mg m^−^^3^
Monoclinic, *P*2_1_/*n*	Mo *K*α radiation, λ = 0.71073 Å
*a* = 3.7978 (4) Å	Cell parameters from 1701 reflections
*b* = 10.5567 (6) Å	θ = 3.0–28.1°
*c* = 18.0366 (10) Å	µ = 5.63 mm^−^^1^
β = 90.208 (7)°	*T* = 173 K
*V* = 723.12 (10) Å^3^	Plate, colourless
*Z* = 4	0.32 × 0.16 × 0.08 mm

### Data collection

**Table:**

Agilent Xcalibur (Ruby, Gemini ultra) diffractometer	1594 independent reflections
Radiation source: Enhance (Mo) X-ray Source	1309 reflections with *I* > 2σ(*I*)
Graphite monochromator	*R*_int_ = 0.040
Detector resolution: 10.3575 pixels mm^-1^	θ_max_ = 29.1°, θ_min_ = 3.0°
ω scans	*h* = −4→3
Absorption correction: multi-scan (*CrysAlis PRO*; Agilent, 2012)	*k* = −12→10
*T*_min_ = 0.094, *T*_max_ = 1	*l* = −21→19
4627 measured reflections	

### Refinement

**Table:**

Refinement on *F*^2^	Primary atom site location: iterative
Least-squares matrix: full	Secondary atom site location: difference Fourier map
*R*[*F*^2^ > 2σ(*F*^2^)] = 0.040	Hydrogen site location: inferred from neighbouring sites
*wR*(*F*^2^) = 0.100	H atoms treated by a mixture of independent and constrained refinement
*S* = 1.08	*w* = 1/[σ^2^(*F*_o_^2^) + (0.0507*P*)^2^ + 0.2934*P*] where *P* = (*F*_o_^2^ + 2*F*_c_^2^)/3
1594 reflections	(Δ/σ)_max_ < 0.001
108 parameters	Δρ_max_ = 1.19 e Å^−^^3^
2 restraints	Δρ_min_ = −0.79 e Å^−^^3^

### Special details

**Table:**

Geometry. All e.s.d.'s (except the e.s.d. in the dihedral angle between two l.s. planes) are estimated using the full covariance matrix. The cell e.s.d.'s are taken into account individually in the estimation of e.s.d.'s in distances, angles and torsion angles; correlations between e.s.d.'s in cell parameters are only used when they are defined by crystal symmetry. An approximate (isotropic) treatment of cell e.s.d.'s is used for estimating e.s.d.'s involving l.s. planes.

### Fractional atomic coordinates and isotropic or equivalent isotropic displacement parameters (Å^2^)

**Table:**

	*x*	*y*	*z*	*U*_iso_*/*U*_eq_	
Br1	0.28846 (10)	0.31635 (3)	0.19518 (2)	0.02717 (17)	
O2	0.9783 (9)	0.1747 (2)	0.51138 (16)	0.0323 (7)	
H2	1.055 (16)	0.109 (4)	0.535 (3)	0.08 (2)*	
O1	0.5137 (8)	0.1225 (2)	0.30358 (15)	0.0304 (7)	
H1	0.574 (15)	0.064 (4)	0.334 (3)	0.060 (16)*	
O3	0.7802 (8)	0.0380 (2)	0.42729 (15)	0.0330 (7)	
C3	0.4674 (10)	0.3437 (3)	0.2917 (2)	0.0214 (8)	
C2	0.5652 (10)	0.2381 (3)	0.3340 (2)	0.0216 (8)	
C1	0.7081 (10)	0.2576 (3)	0.4042 (2)	0.0214 (8)	
C7	0.8233 (10)	0.1476 (4)	0.4484 (2)	0.0241 (8)	
C4	0.5058 (10)	0.4649 (3)	0.3191 (2)	0.0251 (8)	
H4	0.4369	0.5356	0.2899	0.030*	
C6	0.7467 (10)	0.3806 (3)	0.4318 (2)	0.0242 (8)	
H6	0.8432	0.3935	0.4799	0.029*	
C5	0.6454 (11)	0.4832 (4)	0.3896 (2)	0.0284 (9)	
H5	0.6711	0.5666	0.4087	0.034*	

### Atomic displacement parameters (Å^2^)

**Table:**

	*U*^11^	*U*^22^	*U*^33^	*U*^12^	*U*^13^	*U*^23^
Br1	0.0292 (3)	0.0333 (3)	0.0190 (3)	0.00063 (15)	−0.00907 (17)	0.00363 (14)
O2	0.049 (2)	0.0296 (16)	0.0187 (16)	0.0017 (12)	−0.0129 (14)	0.0054 (11)
O1	0.0443 (19)	0.0232 (15)	0.0234 (16)	0.0006 (12)	−0.0131 (13)	0.0003 (11)
O3	0.049 (2)	0.0252 (15)	0.0250 (16)	0.0053 (12)	−0.0134 (13)	0.0000 (11)
C3	0.019 (2)	0.034 (2)	0.0118 (19)	0.0021 (15)	−0.0018 (15)	0.0026 (14)
C2	0.0177 (19)	0.027 (2)	0.020 (2)	−0.0001 (14)	−0.0004 (15)	0.0025 (14)
C1	0.0155 (19)	0.030 (2)	0.018 (2)	0.0012 (14)	0.0004 (15)	0.0031 (14)
C7	0.022 (2)	0.035 (2)	0.015 (2)	0.0033 (15)	−0.0009 (16)	0.0018 (15)
C4	0.023 (2)	0.027 (2)	0.026 (2)	0.0009 (15)	−0.0025 (17)	0.0022 (15)
C6	0.026 (2)	0.028 (2)	0.018 (2)	−0.0012 (15)	−0.0051 (16)	−0.0004 (14)
C5	0.033 (2)	0.020 (2)	0.031 (2)	−0.0008 (15)	−0.0031 (18)	−0.0001 (14)

### Geometric parameters (Å, º)

**Table:**

Br1—C3	1.889 (4)	C2—C1	1.391 (5)
O2—C7	1.309 (5)	C1—C6	1.398 (6)
O2—H2	0.87 (2)	C1—C7	1.474 (5)
O1—C2	1.352 (5)	C4—C5	1.388 (6)
O1—H1	0.85 (2)	C4—H4	0.9500
O3—C7	1.229 (5)	C6—C5	1.379 (5)
C3—C4	1.379 (5)	C6—H6	0.9500
C3—C2	1.400 (5)	C5—H5	0.9500
			
C7—O2—H2	114 (4)	O3—C7—C1	122.4 (3)
C2—O1—H1	111 (4)	O2—C7—C1	115.4 (3)
C4—C3—C2	121.0 (4)	C3—C4—C5	119.8 (3)
C4—C3—Br1	120.7 (3)	C3—C4—H4	120.1
C2—C3—Br1	118.3 (3)	C5—C4—H4	120.1
O1—C2—C1	123.9 (3)	C5—C6—C1	120.3 (4)
O1—C2—C3	117.4 (3)	C5—C6—H6	119.9
C1—C2—C3	118.7 (3)	C1—C6—H6	119.9
C2—C1—C6	120.1 (3)	C6—C5—C4	120.1 (3)
C2—C1—C7	119.3 (3)	C6—C5—H5	120.0
C6—C1—C7	120.6 (3)	C4—C5—H5	120.0
O3—C7—O2	122.2 (3)		
			
C4—C3—C2—O1	178.9 (4)	C6—C1—C7—O3	176.9 (4)
Br1—C3—C2—O1	−2.2 (5)	C2—C1—C7—O2	175.1 (3)
C4—C3—C2—C1	−1.0 (6)	C6—C1—C7—O2	−3.8 (5)
Br1—C3—C2—C1	177.9 (3)	C2—C3—C4—C5	0.4 (6)
O1—C2—C1—C6	−178.9 (3)	Br1—C3—C4—C5	−178.5 (3)
C3—C2—C1—C6	1.0 (5)	C2—C1—C6—C5	−0.3 (6)
O1—C2—C1—C7	2.1 (6)	C7—C1—C6—C5	178.6 (4)
C3—C2—C1—C7	−178.0 (3)	C1—C6—C5—C4	−0.3 (6)
C2—C1—C7—O3	−4.2 (6)	C3—C4—C5—C6	0.3 (6)

### Hydrogen-bond geometry (Å, º)

**Table:**

*D*—H···*A*	*D*—H	H···*A*	*D*···*A*	*D*—H···*A*
O1—H1···O3	0.85 (2)	1.88 (4)	2.604 (4)	142 (5)
O2—H2···O3^i^	0.87 (2)	1.80 (2)	2.664 (4)	172 (6)

Symmetry code: (i) −*x*+2, −*y*, −*z*+1.
