# Supplementary material for: Crystal structure of 3-bromo-2-hy­droxy­benzoic acid
Source: Acta Crystallogr E Crystallogr Commun. 2015 Apr 22;71(Pt 5):531–5. doi: 10.1107/S2056989015007331 (PMC4420063; doi:10.1107/S2056989015007331)
Supplement: Supplementary file 3 [file e-71-00531-Isup3.pdf]

Supporting information

Crystal structure of 3-bromo-2-hydroxybenzoic acid

Gerhard Laus,[a] Volker Kahlenberg,[b] Thomas Gelbrich,[a]* Sven Nerdinger[c] and Herwig Schottenberger[a]

[a]University of Innsbruck, Faculty of Chemistry and Pharmacy, Innrain 80, 6020 Innsbruck, Austria, [b]University of Innsbruck, Institute of Mineralogy and Petrography, Innrain 52, 6020 Innsbruck, Austria, and [c]Sandoz GmbH, Biochemiestrasse 10, 6250 Kundl, Austria

Correspondence e-mail: thomas.gelbrich@uibk.ac.at

S1. Results of *XPac* comparisons

Table S1. Overview of the investigated substituted derivatives of salicylic acid and results of an *XPac* study aimed at identifying common packing motifs with the reference structure of 3-Br.

Compound	Short notation	Refcode [a]	SC [b]	x_{10} [c]	*d* (Å) [d]
3-Bromo-2-hydroxybenzoic acid	3-Br	–	*reference*		3.80
Salicylic acid	SalAc	SALIAC17	**A0**	9.7	.
3,4-difluoro-2-hydroxybenzoic acid	3,4-F	HIXPAH	**A0**	6.1	.
2,3,4-Trihydroxybenzoic acid hydrate	3,4-OH · 0.25H_2O	LAPZUZ	**A11**	5.6	3.73
2,3,4-Trihydroxybenzoic acid dihydrate	3,4-OH · 2H_2O	MUQHOW	none	.	.
2,3,5-Trichloro-6-hydroxybenzoic acid	3,5,6-Cl	MIQKEE02	**A0**	10.1	.
3,5-Dibromo-2-hydroxybenzoic acid	3,5-Br	XISGEM	**S2**	11.8	.
3,5-Dichloro-2-hydroxybenzoic acid	3,5-Cl	WECXAE	**S2**	12.4	.
3,5-Dichloro-2-hydroxy-4-methoxy-6-n-propylbenzoic acid	3,5-Cl, 4-OMe, 6-*n*Pr	COPJAS	**A0**	10.3	.
2-Hydroxy-3,5-diisopropylbenzoic acid	3,5-*i*Pr	NEQTAF	**A0**	7.0	.
3,5-Dinitrosalicylic acid monohydrate	3,5-NO_2 · H_2O (E)	ZAJGUM	none	.	.
3,5-Dinitrosalicylic acid monohydrate	3,5-NO_2 · H_2O (F)	ZAJGUM01	none	.	.
3,5-Di-t-butyl-2-hydroxybenzoic acid	3,5-*t*Bu	ELIDUY	**A0**	4.3	.
3-Butyl-2-hydroxybenzoic acid	3-Bu	HEWLUS	**A0**	6.9	.
3-(Ethoxycarbonyl)-2-hydroxy-6-methoxy-4-methylbenzoic acid	3-COOEt, 4-Me, 6-OMe	ZAPYEW	**X11**	11.1	3.72
2-Hydroxy-1,3-dicarboxybenzene	3-COOH	IBUMAT	none	.	.
3-Carboxysalicylaldehyde monohydrate	3-CHO · H_2O	JOHXEJ	none	.	.
2-Hydroxybenzene-1,2-dioic acid monohydrate	3-COOH · H_2O	TOLCAY02	**X11**	16.4	3.67
2-Hydroxy-3-methylbenzoic acid	3-Me	CRESOT10	**A11**	16.7	4.11
2-Hydroxy-6-isopropyl-3-methylbenzoic acid	3-Me, 6-*i*Pr	URAKUU	**A0**	7.9	.
2-Hydroxy-3-nitrobenzoic acid monohydrate	3-NO_2 · H_2O	VAXXID	**X11**	15.9	3.59
2,3-Dihydroxybenzoic acid	3-OH (triclinic)	CACDAM01	**A0**	8.0	.
2,3-Dihydroxybenzoic acid	3-OH (monoclinic)	CACDAM	none	.	.
2-Hydroxy-3-methoxybenzoic acid	3-OMe	PIDJES01	**A11**	16.1	3.79
2-Hydroxy-3-methoxybenzoic acid monohydrate	3-OMe · H_2O	DIWNON01	none	.	.
5-Methyl-3-sulfosalicylic acid dihydrate	3-SO3, 5-Me · 2H_2O	MSUSAL	**A0**	3.8	.
2,4,6-Trihydroxybenzoic acid monohydrate	4,6-OH · H_2O	XIPVEY	none	.	.
4-Acetamido-2-hydroxybenzoic acid	4-ACM	VAXXOJ	none	.	.
4-Chloro-2-hydroxybenzoic acid	4-Cl	VAXYAW	**A11**	9.2	3.72
2,5-Dihydroxybenzene-1,4-dicarboxylic acid dihydrate	4-COOH, 5-OH · 2H_2O	DUSJUX	none	.	.
2-Hydroxy-4-methylbenzoic acid	4-Me	VAXYIE	**A11**	11.5	3.87
4-Amino-2-hydroxybenzoic acid	4-NH_2	AMSALA02	**A11**	10.3	3.73
5-Carboxy-2,4-dihydroxyanilinium chloride	4-NH_3^+, 5-COOH · Cl^-	SUZTAJ	**A0**	8.3	.
2,4-Dihydroxybenzoic acid	4-OH (I)	ZZZEEU08	**A0**	4.0	.
2,4-Dihydroxybenzoic acid	4-OH (II)	ZZZEEU04	**A11**	10.7	3.69
2,4-Dihydroxybenzoic acid hemihydrate	4-OH · 0.5H_2O	QIVTUK01	**A0**	7.6	.
6-n-Pentadecyl-2,4-dihydroxy-benzoic acid	4-OH, 6-*n*Pentadecyl	PDCHBZ10	**A0**	9.7	.
2-Hydroxy-4-methoxybenzoic acid	4-OMe	VAXYEA	**A0**	4.3	.
5-Acetamido-2-hydroxybenzoic acid monohydrate	5-ACM · H_2O	VAXYOK	**X11**	2.2	3.75
5-Bromo-2-hydroxybenzoic acid	5-Br (α)	IYAWIO01	**A0**	7.7	.
5-Bromo-2-hydroxybenzoic acid	5-Br (β)	IYAWIO02	**A0**	11.4	.
5-Formyl-2-hydroxybenzoic acid	5-CHO	UJOFEF	**X11**	5.6	3.78
5-Chloro-2-hydroxybenzoic acid	5-Cl	VABVAX01	**A11**	7.1	3.71
5-Chloro-2-hydroxybenzoic acid monohydrate	5-Cl · H_2O	VAYBOO	**X11**	13.1	3.73
4-Hydroxyisophthalic acid	5-COOH	OJICEP	**A11**	4.8	3.68
5-Fluorosalicylic acid	5-F	ABENEB	**A11**	4.2	3.82
2-Hydroxy-5-iodobenzoic acid	5-I (α)	VAXZIF	**A11**	14.8	4.58
2-Hydroxy-5-iodobenzoic acid	5-I (β)	VAXZIF01	**A0**	7.7	.
2-Hydroxy-5-methylbenzoic acid	5-Me	BESKEP01	**A0**	6.0	.
5-Ammonio-2-hydroxybenzoate	5-NH_3^+	SAQJAV01	**X11**	20.5	3.72
5-Nitrososalicylic acid	5-NO	NTSALA	**A11**	9.5	3.67
2-Hydroxy-5-nitrobenzoic acid	5-NO_2	GUTNIS01	**A0**	5.9	.
2,5-Dihydroxybenzoic acid	5-OH (I)	BESKAL02	**A0**	4.4	.
2,5-Dihydroxybenzoic acid	5-OH (II)	BESKAL03	**A0**	5.2	.
2-Hydroxy-5-methoxybenzoic acid	5-OMe	VAXZUR	**A11**	5.6	3.98
2-Fluoro-6-hydroxybenzoic acid	6-F	VAYBUU	**A0**	5.5	.
2,6-Dihydroxybenzoic acid	6-OH (orthorhombic)	LEZJAB	**X11**	13.8	3.83
2,6-Dihydroxybenzoic acid	6-OH (monoclinic)	LEZJAB01	**A0**	6.6	.
2,6-Dihydroxybenzoic acid monohydrate	6-OH · H_2O	LEZJEF	none	.	.
2-Hydroxy-6-methoxybenzoic acid	6-OMe	VAYCAB	none	.	.

[a] CSD Refcode. [b] Largest supramolecular construct shared with 3-Br. [c] Dissimilarity index x_{10} for the largest supramolecular construct shared with 3-Br. [d] Length of the stacking vector of the SCs **X11** / **A11**.

Table S2. Crystallographic parameters associated with the occurrence of the two-periodic SC **S2** (Figure 3a) in three crystal structures.

Crystal structure	Refcode [a]	Corresponding lattice parameters [b]						x_{10} [c]
		$\mathbf{t}_1$	d_1 (Å)	$\mathbf{t}_2$	d_2 (Å)	$\angle$ ($\mathbf{t}_1$, $\mathbf{t}_2$)	plane	
3-Br	–	010	10.56	301	21.30	90	$(10\bar{3})$	–
3,5-Br	XISGEM	010	11.08	201	22.65	90	$(10\bar{2})$	11.8
3,5-Cl	WECXAE	101	10.93	$0\bar{1}0$	21.63	90	$(10\bar{1})$	12.4

[a] CSD Refcode. [b] $\mathbf{t}_1$, $\mathbf{t}_2$ = lattice directions; d_1, d_2 = length of translation along $\mathbf{t}_1$ and $\mathbf{t}_2$, respectively; $\angle$ ($\mathbf{t}_1$, $\mathbf{t}_2$) = angle between $\mathbf{t}_1$ and $\mathbf{t}_2$; plane = plane defined by $\mathbf{t}_1$ and $\mathbf{t}_2$. [c] Dissimilarity index x_{10} for supramolecular construct S2 for the comparison with 3-Br.
